# Transformation of Teamwork and Leadership into Obstetric Safety Culture with Crew Resource Management Programme in a Decade

**DOI:** 10.3390/healthcare13202564

**Published:** 2025-10-11

**Authors:** Eric Hang-Kwong So, Victor Kai-Lam Cheung, Ching-Wah Ng, Chao-Ngan Chan, Shuk-Wah Wong, Sze-Ki Wong, Martin Ka-Wing Lau, Teresa Wei-Ling Ma

**Affiliations:** 1 Multi-Disciplinary Simulation and Skills Centre (MDSSC), Queen Elizabeth Hospital, Hong Kong SAR, China; sohke@ha.org.hk; 2 School of Health Sciences, The University of Manchester, Manchester M13 9PL, UK; 3 Department of Obstetrics & Gynaecology (O&G), Queen Elizabeth Hospital, Hong Kong SAR, China

**Keywords:** crew resource management, obstetrics and gynaecology, human factors, conflict resolution, safety culture

## Abstract

In parallel with technical training on knowledge and skills of task-specific medical or surgical procedures, wide arrays of soft skills training would contribute to obstetric safety in the contemporary healthcare setting. This article, as a service evaluation, explored the effect of a specialty-based Crew Resource Management (CRM) training series that transforms the concept of human factors into sustainable measures in fostering clinical safety culture of the Department of Obstetrics and Gynaecology (O&G) in the Queen Elizabeth Hospital. Within the last decade, a tri-phasic programme has been implemented by an inter-professional workgroup which consists of a consultant anaesthesiologist, medical specialists and departmental operations manager from O&G, a nurse simulation specialist, hospital administrators, and a research psychologist. (1) Phase I identified different patterns of attitudinal changes (in assertiveness, communication, leadership, and situational awareness, also known as “ACLS”) between doctors and nurses and between generic and specialty-based sessions for curriculum planning. (2) Phase II evaluated how these specific behaviours changed over 3 months following CRM training tailored for frontline professionals in O&G. (3) Phase III examined the coping style in conflict management and the level of sustainability in self-efficacy over 3 months following specialty-based CRM training. The findings showed the positive impacts of O&G CRM training on healthcare professionals’ increased attitude and behaviour in “ACLS” by 22.7% at a *p* < 0.05 level, character strengths in conflict management, and non-inferior or sustained level of self-efficacy under tough conditions in the clinical setting up to 3 months after training. As a way forward, incorporating a scenario-based O&G CRM programme into existing skills-based training is expected to change service framework with an innovative approach. In addition, exploring actual clinical outcomes representing a higher level of organisational impacts can be a strategic direction for further studies on the effect of this practical and educational approach on obstetric safety culture.

## 1. Introduction

In the modern healthcare environment, ensuring obstetric safety is of paramount importance. Being aligned with global consensus in strengthening clinical safety through training, Crew Resource Management (CRM) has been adopted as one of the critical tools in fostering a safety culture through Inter-Professional Education (IPE) by the Hospital Authority [1]. To fill the knowledge gap, reviewing these programmes may strengthen our understanding about how attitudinal and behavioural changes in clinical human factors (in assertiveness, communication, leadership, situational awareness) as well as the level of sustainability of character strengths in conflict resolution and self-efficacy up to 3 months after the training [2]. This report synthesises evidence-based findings from tri-phasic studies carried out at the Multi-Disciplinary Simulation and Skills Centre (MDSSC) in the Queen Elizabeth Hospital (QEH) within a decade. Each study evaluates the impact of specialised CRM training on attitudes, behaviours, and professional practices of staff members from the Department of Obstetrics and Gynaecology (O&G) [3,4,5].

## 2. Phase I: Transformative Changes in Attitudes Towards Human Factors (2016–2018)

Since 2016, specialised CRM training has marked a significant shift in how healthcare professionals approach optimal teamwork and clinical safety [2].

### 2.1. Objectives

The first study aimed to (i) evaluate the change in participants’ attitudinal shift in human factors, including assertiveness, communication, leadership and followership, and situational awareness (also known as “ACLS”) following training and (ii) identify different patterns of attitudinal changes by different professional roles (e.g., doctor vs. nurse) and by classification of curriculum planning (e.g., generic vs. specialty-based) [2].

### 2.2. Methods

#### 2.2.1. Procedures

A workgroup was steered by a consultant anaesthesiologist, medical specialists and departmental operations manager from O&G, a nurse simulation specialist, hospital administrators, and a research psychologist to maintain quality assurance in the training curriculum, ensure that teaching objectives aligned with training needs, and produce an evaluation encompassing the effect of training and its direction for advancement in medical education [5]. Without restrictive sampling methods, the recruitment of participants was based on convenience sampling, accepting the nomination of medical or nursing staff from designated department heads or designated ward managers from all clinical departments of QEH.

#### 2.2.2. Participants

Of 168 participants from 12 CRM training classes, one-fourth were nominated from the Department of O&G. Female–male and nurse–doctor ratios were distributed at 8:2, approximately.

#### 2.2.3. Measures and Instruments

Human Factor Analysis Survey (HFAS), a 22-item Likert scale with excellent content validity and inter-item reliability (Cronbach’s α = 0.89), was self-reported by participants after training [2,5].

#### 2.2.4. Data Analysis

A comparison of means was performed using the *t*-test for independent samples, using whether participants were nurses or doctors (as the primary outcome) as a comparison variable, with Dunnett’s post hoc tests performed on different specialties, with particular focus on the O&G class compared with the generic class (as the secondary outcome).

### 2.3. Results and Interpretation

**Figure 1:**
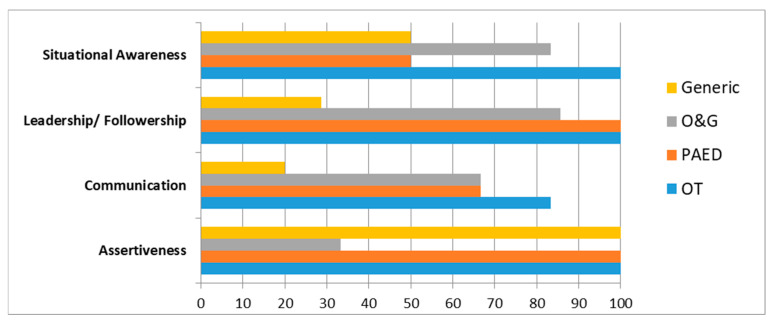
Percentage of items reaching significant attitudinal changes for four human factor components.

Based on the independent t and Dunnett’s post hoc tests, doctors (M = 0.30, SD = 0.32, with a 95% CI [0.185, 0.415]) and nurses (M = 0.227, SD = 0.252, with a 95% CI [0.181, 0.269]) showed no statistical difference in overall changes in HFAS (t(166) = −1.18, *p* = 0.24, two-tailed, with a 95% CI [−0.050, 0.196]). In reviewing significant changes in the proportion of HFAS items, nurses outweighed doctors by 46%, and it was noted that a “leader should take into account team member’s concerns for a decision” was identified as the most invaluable change in team work of obstetric service throughout the O&G CRM programme (pre- and post-*difference* = 0.40, *p* = 0.03, with a 95% CI [0.28, 0.44]) (see Figure 1 and Table 1).

With consistent findings of HFAS in 2016, CRM showed promising results on positive attitudinal changes in clinical human factors, particularly for nurses [2,5]. This finding may be due to the imbalanced doctor–nurse ratio (1:4.6), or distinctive preference for type of training (e.g., doctors had a preference for training in specific medical or surgical procedures with hands-on practice, while nurses had a preference for training in communication skills and management process closer to the nature of CRM programme). Compared with generic programmes, O&G specialty-based programmes resulted in an additive impact on overall attitudinal changes in participants from the Department of O&G by 22.7%, especially on leadership (57.1%) and situational awareness (33.3%). The finding supported the notion that replacing “one-size-fit-all” training curricula with department-specific modifications could optimise the effects of training on ACLS, in turn fostering obstetric safety culture to a larger extent.

## 3. Phase II: O&G Specialty-Based Training and Behavioural Shifts (2018–2021)

### 3.1. Objectives

Following the established evidence of positive learning effects at the end of the specialty-based CRM training, the next study aimed to (i) apply a reliable and valid tool for evaluating specific behaviours and (ii) assess the extent to which the specialty-based CRM of O&G produces positive change in established behavioural components over the 3 months following the training [5].

### 3.2. Methods

#### 3.2.1. Procedures

As a specialty-based training, limited seats were offered to any medical and nursing staff members who were nominated by the department head or designated ward managers from the Department of O&G of QEH.

#### 3.2.2. Participants

All participants nominated by supervisors, including 10 physicians and 59 nurses, were invited to complete the outcome measures.

#### 3.2.3. Measures and Instruments

The decision to retire the Human Factor Analysis Survey (HFAS) was based on comments from an evaluation meeting in 2018, highlighting its drawbacks: (a) long sentences, (b) double-barrelled questions, and (c) direct adoption from a previous study with low-validity for specific elements. A Self-Evaluated Behaviour Assessment (SEBA) was developed to establish a reliable and valid measure to assess specific behavioural aspects of human factors (e.g., assertiveness, communication, leadership, followership, and situational awareness) for further use [2,5]. The SEBA-28, the 28-item version of SEBA, was validated and endorsed by a multi-disciplinary workgroup (comprising an O&G consultant, consultant anaesthetist, nurse consultant, ward manager, advanced practice nurses, and an executive officer), with universal agreement on all items and excellent internal consistency (Cronbach’s alpha = 0.95; ranged from 0.87 to 0.91 for all subscales) [5].

#### 3.2.4. Data Analysis

All participants nominated by supervisors were invited to fill out the SEBA-28 at two time points: (1) at the end of the training (pre) and (2) 3 months after training (post). Anonymised crude data were analysed by paired-sample *t*-tests, with unequal variance assumed, with IBM SPSS Statistics (version 25).

### 3.3. Results and Interpretation

**Figure 2:**
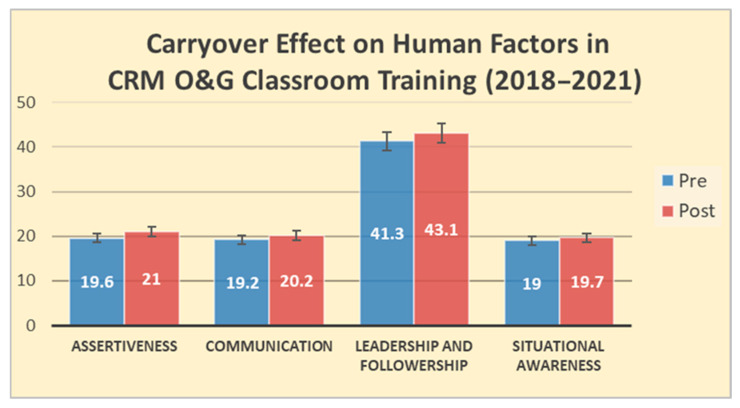
Carryover effect on human factors in CRM O&G classroom training.

A total of 5 classes trained 10 doctors and 59 nurses from the Department of O&G from 2018 to 2021. At 3 months after training, significant changes were found in all sub-scores of assertiveness (mean difference = 1.33, 95% CI [0.83, 1.84]), communication (mean difference = 0.97, 95% CI [0.33, 1.61]) and leadership (mean difference = 1.84, 95% CI [0.66, 3.02]) at *p* < 0.01, of situational awareness (mean difference = 0.61, 95% CI [0.06, 1.16]) at *p* < 0.05, and total scores at *p* < 0.01 (mean difference = 4.75, 95% CI [2.35, 7.16]) (see Table 2 and Figure 2). The findings illustrated translational effect of O&G-specific CRM training on upskilling soft skills related to clinical human factors and patient safety: speak-up, communication, leadership, and situational awareness

## 4. Phase III: Integration of Conflict Management into CRM Training (2021–2024)

### 4.1. Objectives

With evolving needs in the service management and occupation culture of the Department of O&G, since 2021, the scope of CRM has been laterally expanding from traditionally adopted elements in clinical human factors (assertiveness, communication, leadership and followership, situational awareness) to specific conflict management styles and the self-efficacy of healthcare professionals [3,4,5].

### 4.2. Methods

#### 4.2.1. Procedures

Recruitment methods and inclusion criteria for this specialty-based training remained the same as those set out in the previous phase: any medical and nursing staff members who were nominated by the department head or designated ward managers from the Department of O&G of QEH.

#### 4.2.2. Participants

All participants nominated by supervisors, including 17 physicians and 78 nurses, were invited to complete the outcome measures.

#### 4.2.3. Measures and Instruments

**Figure 3:**
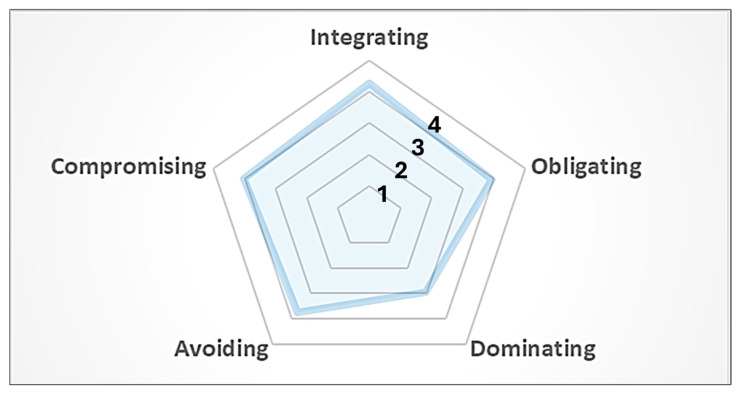
Overall distribution of leadership styles of conflict management from ROCI-II.

In order to examine the level of carryover effect in conflict resolution and coping style and self-efficacy 3 months post-training, two psychometrically sound scales, namely the Rahim Organisational Conflict Inventory (ROCI-II) [6,7] and General/Specific Self-Efficacy (GSE/SSE) Scales [8,9,10] have been incorporated into the curriculum of O&G CRM training since 2021. The ROCI-II measures predominant styles of conflict management by “integrating” (or collaborative), “yielding” (or accommodating), “dominating” (or competitive), “avoiding”, and “compromising” [6,7] (see Figure 3), while the GSE Scales measure how strongly participants believe in their ability to manage various tough situations based on internal resources (Cronbach’s alpha = 0.95, with subscale scores ranging from 0.76 to 0.90) [8,9,10].

#### 4.2.4. Data Analysis

All participants nominated by supervisors were invited to complete the full set of questionnaires (including ROCI-II and GSE/SSE) at two time points: (1) at the end of the training (pre) and (2) 3 months after training (post). Anonymised crude data were analysed by paired-sample *t*-tests, with unequal variance assumed, with IBM SPSS Statistics (version 25). Since demographic information was retrieved from the training record without mapping on anonymized data, no sub-group analysis was carried out.

### 4.3. Results and Interpretation

From 2021 to 2024, 9 classes of O&G CRM trained 17 doctors (18%) and 78 nurses (82%), of which the majority were female (98%) with either less than 3 years or over 10 years of specialty experience (68%) in the Department of O&G. Participants were likely to handle peer conflicts with the “integrating” approach (4.3 out of 5) as opposed to the “dominating” approach (2.9 out of 5), with a moderate level of self-efficacy in the general situation (GSE = 27.5 to 27.6 out of 40) and in specific themes on people-centred care (SSE = 22.5 out of 32). Non-significant results demonstrated carryover effects on or non-inferiority to all factors (*p* > 0.05, for all) (see Table 3). In other words, the results without drops at a significant level proved that the O&G CRM programme was effective, resulting in a sustained level of facilitative conflict management styles and self-efficacy up to 3 months post-training.

## 5. Way Forward: Impact on Clinical Safety and Team Dynamics (2025 and Beyond)

This article has demonstrated how the tri-phasic implementation of specialised CRM training could transform teamwork and leadership into sustainable measures, fostering a clinical safety culture in the O&G department [2,3,4,5]. Collaborating alongside subject-matter experts from both clinical departments and medical simulation training centres enabled healthcare professionals in either frontline or management roles to gain actionable insights into the potential effect of safety factors on teams. Through the CRM training with carefully designed components (e.g., interactive and ice-breaking games, high-stakes obstetrics scenarios, self-exploration on character strengths and coping strategies…, etc.), participants could reflect on personal experience in handling challenging cases related to intricate group dynamics and acknowledge the genuine needs of positive shifts in professional attitudes and behaviours in core competencies for technical aspects or task-specific procedures [3,4].

In addition to cultivating clinical human factors on patient safety, incorporating concepts of conflict resolution management and its leadership approach into existing curriculum of O&G CRM training signified the ground-breaking move in addressing evolving dynamics of healthcare service demands. The high quality standards of the healthcare service can be achieved through strengthening continuous education with an up-to-date CRM curriculum, with further research needed on the translational impacts of CRM training on patient safety culture and clinical outcomes following a change in service framework and an innovative approach (e.g., practice of new protocol, use of virtual reality or 3D-printed part-task trainers in hysteroscopy,…, etc.) [5].

## 6. Conclusions

This service evaluation reconfirms the importance of applying concepts of crew resource management to a tri-phasically implemented training program tailored to frontline professionals in the Department of Obstetrics and Gynaecology of the Queen Elizabeth Hospital. The results from Phase I and Phase II showed the positive impact of these specialty-based trainings on cultivating attitudinal and behavioural changes in clinical human factor elements that are highly relevant to patient safety, including assertiveness, communication, leadership (and followership), and situational awareness. In addition, the results from Phase III provided preliminary evidence on how a specialty-based programme for conflict resolution could lead to a sustained level of self-efficacy in the related clinical service over 3 months after training. In addition to all the factors covered in current paper, outcome measurements in relations to actual clinical outcomes (e.g., reduced adverse events, improved maternal safety metrics) and staff well-being statistics (e.g., burnout, retention rates) should be taken into consideration for scaled-up studies with a comprehensive analysis of the effectiveness of training in terms of organisational impact at a higher level.

## Figures and Tables

**Table 1:** Attitudinal changes of four human factor components by specialty or type of professional.

Categories		Assertiveness (3 Items)	Communication (6 Items)	Leadership/ Followership (7 Items)	Situational Awareness (6 Items)	Overall (22 Items)
**All (*n* = 168)**		100%	100%	100%	100%	100%
**By Specialty of CRM Class**	**OT (*n* = 42)**	100%	83.3%	100%	100%	95.5%
	**PAED (*n* = 63)**	100%	66.7%	100%	50%	77.3%
	**O&G (*n* = 31)**	33.3%	66.7%	85.7%	83.3%	72.7%
	**Generic (*n* = 32)**	100%	50%	28.6%	50%	50%
**By Type of Professional**	**Doctor (*n* = 30)**	100%	33.3%	57.1%	50%	54.5%
	**Nurse (*n* = 138)**	100%	100%	100%	100%	100%

Note: OT = operating theatre; Paed = paediatrics; O&G = obstetrics and gynaecology; % indicated proportion of items reaching significant changes after training at *p* < 0.05 level.

**Table 2:** Pre-and-post results of the Self-Evaluated Behaviour Assessment (SEBA-28).

Self-Evaluated Behaviour Assessment (SEBA-28)	2018/19		2019/20		2020/21		Total	
	Pre Test M ± SD	Post Test M ± SD	Pre Test M ± SD	Post Test M ± SD	Pre Test M ± SD	Post Test M ± SD	Pre Test M ± SD	Post Test M ± SD
**Total Score** ***(r*** **= 0.953)**	**102.8** **±** **11.0**	* 110.3 **±** **8.2**	**103.9 ± 7**	**105.1 ± 9.4**	**96.5 ± 11.3**	* **105.1 ± 6.2**	**99.2 ± 9.9**	** **103.9 ± 8.2**
*Assertiveness* ***(r*** **= 0.859)**	19.1 ± 1.9	* 20.6 ± 1.5	20.5 ± 1.9	* 21.2 ± 1.7	18.9 ± 2.1	** 21.1 ± 2.0	19.6 ± 2.1	** 21.0 ± 1.7
*Communication* ***(r*** **= 0.874)**	18.3 ± 2.2	* 20.0 ± 1.6	20.3 ± 1.9	20.3 ± 2.1	18.7 ± 2.1	* 20.3 ± 1.2	19.2 ± 2.3	** 20.2 ± 1.7
*Leadership and Followership* ***(r*** **= 0.905)**	39.9 ± 4.4	* 42.3 ± 3.9	43.2 ± 3.7	43.7 ± 4.8	40.0 ± 6.0	* 43.8 ± 3.7	41.3 ± 4.6	** 43.1 ± 4.3
*Situational Awareness* ***(r*** **= 0.888)**	18.2 ± 2.7	* 19.3 ± 2.0	19.9 ± 2.1	19.9 ± 2.3	18.9 ± 2.1	19.8 ± 1.0	19.0 ± 2.4	* 19.7 ± 2.0

**Note: Perceived applicability:** (Pre) 4.01 ± 0.65, (post) 4.23 ± 0.55, NS; **Enhanced Confidence:** (Pre) 3.96 ± 0.65, (post) 4.13 ± 0.57; not significant * *p* < 0.5, ** *p* < 0.01.

**Table 3:** Pre-and-post comparison table of outcome measurements by years.

*n* = 95		2021/22		2022/23		2023/24		2021–2024	
ROCI-II	Highest Score	Pre Test M ± SD	Post Test M ± SD	Pre Test M ± SD	Post Test M ± SD	Pre Test M ± SD	Post Test M ± SD	Pre Test M ± SD	Post Test M ± SD
Integrating	5	4.31 ± 0.47	4.30 ± 0.57	4.27 ± 0.55	4.37 ± 0.55	4.16 ± 0.59	4.22 ± 0.60	4.25 ± 0.54	4.31 ± 0.57
Obliging	5	4.06 ± 0.60	3.96 ± 0.59	3.98 ± 0.57	3.99 ± 0.62	3.77 ± 0.67	3.83 ± 0.61	3.93 ± 0.61	3.93 ± 0.60
Dominating	5	2.91 ± 0.71	2.65 ± 0.63	2.79 ± 0.61	3.07 ± 0.62	3.07 ± 0.68	3.02 ± 0.60	2.91 ± 0.66	2.95 ± 0.63
Avoiding	5	3.77 ± 0.54	3.62 ± 0.62	3.90 ± 0.54	3.86 ± 0.61	3.57 ± 0.60	3.56 ± 0.59	3.76 ± 0.57	3.71 ± 0.61
Compromising	5	3.96 ± 0.60	3.86 ± 0.69	4.11 ± 0.57	4.10 ± 0.52	3.97 ± 0.59	3.97 ± 0.57	4.03 ± 0.58	4.00 ± 0.59
GSE	40	27.61 ± 0.45	28.57 ± 3.92	26.74 ± 4.07	27.33 ± 4.52	28.50 ± 3.79	27.33 ± 6.38	27.51 ± 4.12	27.63 ± 5.04
SSE	32	24.04 ± 0.28	24.13 ± 3.51	21.79 ± 4.27	21.98 ± 4.19	22.43 ± 3.23	21.93 ± 5.19	22.54 ± 3.88	22.48 ± 4.44

**Note:** ROCI = Rahim Organisational Conflict Inventory; GSE = general self-efficacy; SSE = specific self-efficacy. **Results:** *p* > 0.05; not significant for all, except “Dominating” in 2022/23 using Wilcoxon signed rank tests.

## Data Availability

Since the data from this study are owned by the Queen Elizabeth Hospital and managed in accordance with the data handling policy of the Hospital Authority, the decision of data sharing may be considered on reasonable request, subject to approval.

## Abbreviations

The following abbreviations are used in this manuscript:

CRM	Crew Resource Management
O&G	Obstetrics and Gynaecology
IPE	Inter-Professional Education
QEH	Queen Elizabeth Hospital
ACLS	Assertiveness, Communication, Leadership, and Situational Awareness
OT	Operating Theatre
HFAS	Human Factor Analysis Survey
SEBA-28	Self-Evaluated Behaviour Assessment
ROCI-II	Rahim Organisational Conflict Inventory
GSE	General Self-Efficacy
SSE	Specific Self-Efficacy
